# Molar Incisor Hypomineralization: Prevalence and Risk Factors Among 7-9 Years Old School Children in Muradnagar, Ghaziabad

**DOI:** 10.2174/1745017901814010714

**Published:** 2018-09-28

**Authors:** Archana Rai, Avnish Singh, Ipseeta Menon, Jyoti Singh, Vineet Rai, Gunjan Singh Aswal

**Affiliations:** 1 Public Health Dentistry, Atlas College of Health Sciences, Addis Ababa, Ethiopia; 2 Public Health Dentistry, Seema Dental College, Rishikesh, India; 3 Public Health Dentistry, ITS- CDSR, Muradnagar, Ghaziabad, India; 4 Public Health Dentistry, Uttranchal Dental and Medical Research Institute, Dehradun, India; 5 Department of Conservative Dentistry and Endodontics, Institute of Health Sciences, Jimma University, Jimma, Ethiopia; 6 Department of Prosthodontics, Institute of Health Sciences, Jimma University, Jimma, Ethiopia

**Keywords:** Developmental enamel defects, School children, Molar incisor hypomineralization, Enamel hypoplasia, Demarcated opacity, Diffuse opacity, Modified developmental defect of enamel index, Risk factors

## Abstract

**Background:**

The aim of this school-based, cross-sectional survey was to investigate the prevalence and risk factors of permanent Molar Incisor Hypomineralization among 7-9 years old school children in Muradnagar.

**Methods:**

This cross-sectional study was performed among 7-9 years old school children in Muradnagar. Estimated sample size was 992 in this study. Multistage cluster sampling technique was used in this study in which schools were the clusters which were selected randomly. The study proforma was divided into 2 parts, first part comprised of demographic status, socio-economic status, questionnaires on risk factors of Molar Incisor Hypomineralisation such as prenatal, perinatal and postnal history upto 3 years, feeding pattern, fluoride and other pollutants exposure history, dental history, history of trauma to teeth/face, family history of enamel defects which was asked to the mothers and filled by one examiner in a face to face interview. The second part comprised of recording format of clinical variables assessed by investigator to be recorded by the recording clerk. Molar incisor hypomineralisation were recorded using Modified Developmental Defect of Enamel index developed by Clarkson J.J. and O’ Mullane D.M. in 1989 and dental caries by using Decayed Missing Filled Tooth index (World Health Organization Modification 1997) in which WHO probe was used for examination of dental caries as recommended by WHO in the Oral Health Surveys, Fourth edition; 1997 (Spanish version). Before 1997 modification dental explorer was used for the examination of dental caries. *Chi* square test, Pearson’s Correlation test, Logistic Regression Analysis and Unpaired *t*-test were used for analysing the data.

**Results:**

The overall prevalence rate of Molar incisor hypomineralisation was 21.4% in this study. Age, problems during pregnancy, normal delivery and childhood illness/ infections are the risk factors which have highest strength of association.

**Conclusion:**

In the present study Molar incisor hypomineralisation was found to affect 2 out of every 10 children examined which was higher than that observed in other studies on Indian children.

## 1 INTRODUCTION

Molar Incisor Mineralization (MIH) is a common developmental condition resulting in the developmental enamel defects in first permanent molars and permanent incisors. It appears at the eruption of these teeth. One of the four permanent molars, and often also the incisors, could be affected. Early diagnosis is needed since, rapid breakdown of tooth structure may occur which leads to acute symptoms and complicated treatment.

Enamel defects are known to occur due to depressed activity of the enamel forming ameloblasts due to various risk factors (such as illness, antibiotic use and excessive fluoride intake) during prenatal, perinatal or postnatal period [1] which result in the formation of linearly distributed pits or grooves. These alterations can be found in two different stages: Enamel matrix formation (secretion phase) and enamel mineralization (maturation phase). If an unbalance occurs during the secretion phase, the enamel defect is called hypoplasia [2, 3]. If it occurs during the maturation phase, it is called hypomineralization. Once formed, enamel is not remodeled again during life and every individual’s enamel is a record of the first 8 or 9 years of their life when the crowns are formed [4]. Hypoplasia is associated with the smallest thickness of the affected enamel present as shallow or deep fossae with horizontal or vertical grooves and with partial or total absence of enamel. Hypomineralization presents as an anomaly in the tissue translucency. A white or yellowish/brownish area can be seen on enamel and there is no alteration in thickness. Recently, one enamel alteration affecting the First Permanent Molars (FPM) of great clinical significance was described in four presentations at the European Academy of Pediatric Dentistry Congress in 2000. These reports called the condition hypomineralized FPM [5], idiopathic enamel hypomineralization in FPM [6], nonfluoride hypomineralization in FPM [7] and cheese molars [8]. It was defined as a single clinical entity and termed Molar Incisor Hypomineralization (MIH) in 2001. It is defined as hypomineralization of systemic origin affecting one, two, three or all first permanent molars and the permanent incisors [9]. The severity of MIH may vary greatly. It ranges from mild opacities to posteruptive breakdown. Due to soft and porous enamel of teeth in MIH cases, unusual cavitation and enamel disintegration on the occlusal surface may occur which may cause hypersensitivity, secondary caries, atypical restoration, loss of filling and, in severe cases, extraction of the affected teeth [10, 11]. Anterior teeth anomalies may have a significant psychological impact. In affected incisors, the severity of hypomineralization is usually less than that of the affected molars [8]. Literatures are no more on this highly variable ectodermal disorder, supra added by its manifold clinical features leads to many undiagnosed cases which further lead to an array of clinical errors. MIH has been encountered in clinical practice with reported prevalence between 2.8 percent [12] and 40.2 percent [13] worldwide and 9.2% percent in India [10]. Solution of this problem and possible consequences can be a great challenge for professionals because treatment can be complex for these cases. To address the fact that no study exists regarding prevalence and Risk factors of permanent MIH among 7-9 years old school children in Muradnagar, this study was conducted. The aim of this study is to find out prevalence, putative etiological factors of MIH among 7-9 years old school children in Muradnagar, Ghaziabad.

## 2 MATERIALS AND METHODS

The present school-based cross-sectional survey among 7-9 years old school children of Muradnagar, was conducted during the academic year 2014-2015 after approval of study protocol from Ethical Clearance Committee of I.T.S.-C.D.S.R, Muradnagar in the year 2013. Study setting was Muradnagar in this study. Multi-stage cluster sampling technique was applied to select study population in which schools were the clusters which were selected randomly. In first stage of sampling, Muradnagar was divided into urban and rural area. In the second stage of sampling, government and non- government schools were randomly selected from both urban and rural area till the estimated size of sample (n=992) of study population (7-9 year old School children) was achieved. Randomization technique used in this study was Pick a chit method. Pilot study was carried out to check the feasibility of implementing the study and validity of questionnaire. Face validity and Content validity methods were used for validating the questionnaires.

The sample size was determined based on results of pilot study showing expected prevalence of 12% among 7-9 years old school children. Population of 4,020 of age group 7-9 years old school children were retrieved from Basic Siksha Adhikari. The following formula was used to calculate the sample size:

$$n\;=\;\frac{NZ^{2}\;P(1-P)}{d^{2}(N-1)+Z^{2}P(1-P)}$$

Where, n= Sample size,

N= Population size

Z= Level of confidence (at 95% =1.96),

P = Expected Prevalence (12% =0.12),

d = Precision (2%= 0.02)

According to the above values, sample size required = 810

Keeping 22.5% as non- response rate (which came by pilot study) 182 more subjects have to be taken.

So total sample size required =810+ 182= 992

Sample used in pilot study was not included in the final analysis. Permission for conducting the study was obtained from the principals of the selected schools. Informed written consents were obtained from the parents of the students through school authorities. Study was conducted by the single trained and calibrated examiner from the Department of Public Health Dentistry. Kappa co-efficient was 0.87 in this study. Data collection was done at the day of parent teacher meeting in school to ensure the availability of mothers. Data was collected by completion of the proforma by examiner (investigator) after face to face interview of their mothers in local language Hindi and clinical examination of each child. The study proforma was prepared in both English and local language Hindi. The study proforma was divided into 2 parts, first part covered general information that comprise of demographic status and socio-economic status, questionnaires on risk factors of Molar incisor mineralization such as prenatal, perinatal and postnatal history upto 3 years. The second part comprised of recording format of MIH (using modified Developmental Defect of Enamel (DDE) index developed by Clarkson J.J. and O’ Mullane D.M. in 1989) [14] and dental caries by using Decayed Missing Filled index (DMFT) index (WHO Modification 1997) in which WHO probe was used for examination of dental caries as recommended by WHO in the Oral Health Surveys, Fourth edition; 1997 (Spanish version) [15] assessed by investigator recorded by the recording clerk. Before 1997 modification dental explorer was used for examination of dental caries [16]. The modified DDE index is a descriptive index developed from the DDE index. It covers all the defects based on their macroscopic appearance. It is a more practical and comparable index in epidemiological studies. It is less time consuming, simpler, more specific and meaningful index than the other. Inclusion criteria was children with all first permanent molars and permanent incisors erupted completely in oral cavity, the children in the age group of 7-9 years (*i.e*. after 7^th^ and before 9^th^ birthday as on the day of survey) present in the selected schools on the day of survey, children with permitted participatory informed written consent from the parents, children who were treated for MIH in the past and children who have their biological mother alive. Exclusion criteria was children undergoing orthodontic treatment, children with generalized developmental enamel defects such as Amelogenesis imperfecta and children suffering from a chronic systemic disease. The description and comparison of the data according to study aim and objectives was done using chi square test, Pearson’s Correlation test, Logistic Regression Analysis and Unpaired *t*-test.

## 3 RESULTS

Table **1** shows that a total of 992 School children were examined, out of which 311 (31.35%) belonged to 7-8 years age group and 681 (68.65%) belonged to 8-9 years age group. There were 532 (53.6%) males and 460 (46.4%) females participated in the study. Among 311 School children belonged to 7-8 years age group, 150 (48.2%) were males and 161 (51.8%) were females and among 681 School children belonged to 8-9 years age group, 382 (56.1%) were males and 299 (43.9%) were females.

Graph (**1**) shows that out of 992 school children, 496 (50%) were from urban area and 496 (50%) were from rural area. Among urban area 315 (63.5%) males and 181(36.5%) females were present and among rural area 217 (43.8%) males and 279 (56.2%) females were present.

Graph (**2**) shows that out of 992 school children, 496 (50%) were from Government school and 496 (50%) from Non-Government school. Among Non-Government school 283 (57.1%) males and 213 (42.9%) females participated and among Government schools 249 (51.2%) males and 247 (48.8%) females participated.

Graph (**3**) shows that among 992 school children, in affected MIH cases, 46.2% school children belonged to upper middle class, 37.8% belonged to lower/ upper lower class and16.0% belonged to middle/ lower middle class and in MIH unaffected cases, 48.8% school children belonged to upper middle class, 29.9% belonged to middle/lower middle class and 21.3% belonged to lower/ upper lower class and socioeconomic variable was found highly significant.

Among the 992 school children, total 1984 upper molar teeth were examined. Among the upper molars, 70.5% teeth were normal, 22.8% teeth were found with demarcated opacity, 3.4% were found with diffuse opacity, 1.7% were found with demarcated and diffuse opacity and 1.6% were found with both demarcated opacity and hypoplasia. Among the 992 school children, total of 1984 lower molars were examined. Among the lower molars, 69.8% teeth were found normal, 21.9% with demarcated opacity, 3.4% with diffuse opacity, 3.2% with both demarcated opacity and hypoplasia, 0.9% with demarcated and diffuse opacity and 0.8% with hypoplasia. Among the 992 school children, in 3,968 upper incisors, 6.4% teeth were given the code “not recorded” because more than two-thirds of a tooth surface is heavily restored, badly decayed or fractured. Among the upper incisors, 54.4% teeth were found normal, 23.8% with demarcated opacity, 14.9% with diffuse opacity and 0.5% with demarcated opacity and hypoplasia. Among the 992 School children, in 3,968 lower incisors, 0.8% teeth were given the code “not recorded” because more than two-thirds of a tooth surface is heavily restored, badly decayed or fractured. Among the lower incisors, 72.5% teeth were found normal, 17.3% with demarcated opacity and 9.4% with diffuse opacity.

Logistic regression analysis was done for demographic, prenatal, perinatal and postnatal variables. Among the Demographic variables, School children aged 7-8 years had 5.391 times higher odds of MIH as compared to school children aged 8-9 years. Males had 0.638 times higher odds of MIH than female. School children from lower middle socio-economic status (according to updated Kuppuswamy’s socioeconomic scale for 2012) had 1.763 times higher odds of MIH as compared to upper middle class. School children from upper lower class had 0.534 times higher odds of MIH as compared to upper middle class. All of these were found highly significant. Since, study protocol for this study was approved in 2013, at that time, updated Kuppuswamy’s socioeconomic scale for 2012 was available. So, it was taken for this study.

Among prenatal variables, School children of mothers who had gestational diabetes during pregnancy had 1.291 times higher odds of MIH as compared to those whose mothers were normal. School children of mothers who had used medicines for gestational diabetes had 1.291 times higher odds as compared to those whose mothers did not use them. School children of mothers who had hypertension during pregnancy had 1.304 times higher odds of MIH as compared to those whose mothers did not have. School children of mothers who used B.P. (Blood Pressure) medications during pregnancy had 1.297 times higher odds of MIH as compared to who did not use. School children of mothers who had hypocalcaemia during pregnancy had 1.292 times higher odds of MIH as compared to those whose mothers did not have. School children of mothers who had vitamin D deficiency during pregnancy had 1.292 times higher odds of MIH as compared to those whose mothers did not. All these factors were found highly significant.

Table **2** shows that perinatal variables were also associated with MIH. School children of mothers who had birth complications had 1.304 times higher odds of MIH as compared to those whose mothers did not. School children of mothers who had birth prematurity history had 1.290 times higher odds of MIH as compared to those whose mothers did not. School children of mothers who had excessively prolonged labour pain history had 1.305 times higher odds of MIH as compared to those whose mothers did not. School children of mothers who had normal birth delivery had 2.864 times higher odds of MIH as compared to those whose mothers had cesarean birth delivery. All these factors were found highly significant.

Among postnatal variables School children with childhood illness and infections had 2.641 times higher odds of MIH as compared to those who are healthy. School children with Otitis Media/ Ear infection history had 0.472 times higher odds of MIH as compared to who did not. School children with history of asthma had 1.297 times higher odds of MIH as compared to those who did not. School children with history of asthma medications had 1.312 times higher odds of MIH as compared to those who did not. School children with history of chicken pox had 1.285 times higher odds of MIH as compared to those whose did not. School children with history of allergies had 1.462 times higher odds of MIH as compared to who did not. School children who had history of taking other antibiotics had 0.251 times higher odds of MIH than those who didn’t have. School children who had history of taking other type of medications had 1.290 times higher odds of MIH as compared to who did not. School children who had a history of 6-12 months breast feeding had 2.400 times higher odds of MIH as compared to those who had less than 6 months. All these factors were found significant.

Graph (**4**) shows that MIH was found to be associated with the mean value of decayed teeth 0.37, which was found significant. MIH was found to be associated with the mean value of missing teeth due to caries 0.02 and mean value of DMF 0.39 and these were found highly significant.

## 4 DISCUSSION

This cross-sectional study provides baseline data about prevalence and risk factors of MIH among 7- 9 years old 992 School children studying in government and non-government schools of urban and rural areas in Muradnagar. Relationship of prevalent MIH in study population were compared for association with age, gender, type of school, location of school, prenatal, perinatal, postnatal histories, breast feeding patterns, Fluoride and other pollutants exposure history, dental History, dietary pattern, past history of trauma to teeth/face in first 3 years, family history of Enamel Defect and DMFT score. Recall bias or memory bias was managed in this study as investigator was well trained who defined the research questions carefully and sufficient time was given to every mother for adequate recall of long term memory. Questionnaires were validated by face validity and content validity methods. Family history of enamel defect was asked to recognize genetic disorder and susceptibilities to defect. It acts as a powerful tool that health care providers use especially when laboratory or genetic tests are not available and it helps in individual risk assessment. The modified DDE index (Clarkson and O’Mullane 1989) [14] was used to assess MIH. This index was selected as it was simple to use, provided effective system for recording enamel defects based on clinical criteria and this index was proposed in WHO- Basic Oral Health Survey Manual [15, 16] for recording enamel opacities.

The overall prevalence rate of MIH was found 21.4% similar with the study reported by Ghanim *et al*., [17] (21.5%) in Iraq School children in 7-9 years old School children using EAPD criteria (2003), slightly higher prevalence reported by Arrow P, [18] (22%) in Australia among 6-8 years old School children using modified DDE index. Anjum R, Sudhan ZA, [19] in Jammu, India reported a prevalence of 14.5% in 7-15 year old children coming to dental college using modified DDE index. while Ahmadi R, *et al*., [20] in Iran observed a lower prevalence of 12.7% among 7-9 years old school children using DDE index. The difference in the prevalence of MIH can be explained by different methodology, diagnostic criteria, different age groups and also geographic and cultural variations in the studied populations that influence the type and activities mothers usually practice in their life. Prevalence of MIH was found higher for 7-8 year age group (42.1%) as compared to 8-9 year age group (11.9%) and was highly significant. However, this finding contradicts with the study conducted by Ghanim *et al*., [17], where no significant difference was revealed in the MIH prevalence between different age, Zawaideh *et al*., [21] in which significant relationship between MIH and age was found and higher prevalence was reported in 9 years old as compared to 7 years old. This was due to the presence of specific environmental factors in the year of birth of the group examined.

In this study, prevalence of MIH was higher among males (24.8%) than in females (20.0%) and this was found highly significant. The study conducted by Shrestha R. *et al*. [22] in 7-12 years School children by EAPD criteria 2003, Ahmadi R, Ramazanil N, Nourinasad R [20], and Ghanim *et al*. [17], also reported higher prevalence of MIH in males than in females but this was not significant. But this finding was not similar with the study conducted by Anjum R, Sudhan ZA [19], in Jammu, India, Zawaideh FI *et al* [21]. using EAPD (European academy of pediatric dentistry) criteria established by Weerheijm, 2003 where prevalence was higher among females. This was due to the fact that girls are more advanced than boys in dental development hence affecting first permanent molars. This would expose hypomineralized molars to masticatory forces and leading to the post eruptive breakdown compared with the boys of the same age (Chawla *et al*.,) [11].

Prevalence of MIH was higher among government School children (22.9%) than non-government School children (19.8%). This finding was in favour with the study Robles MJ *et al*., [23] in 3-12 years old School children using modified DDE index (FDI 1992). The reason for such difference might be due to School children going to a government school are an indication of its low socio- economic condition and consequently determine the type of environment where the child lives in.

Prevalence of MIH was higher among urban (22.9%) School children than in rural School children (19.8%) in this study. In my study majority of MIH cases were from lower/ upper lower class (32.5%) followed by upper middle class (20.5%), middle/ lower middle class (12.7%) and this was found highly significant. This finding was in line with the studies conducted by Robles MJ *et al.*, [23]. The reason was different environmental exposure and dietary pattern.

In the study, 262 (26.4%) mothers had history of infections or illness during pregnancy and (25.1%) were MIH affected with history of infection during pregnancy and 20% were MIH affected without history of illness during pregnancy. This was similar to the study conducted by Ahmdi R *et al.*, [20]., The present study revealed that different medical conditions (prenatal, perinatal and postnatal) and environmental factors may be associated with MIH development. This is logical because enamel formation is a long and sensitive process, which may be affected by many environmental changes and also various childhood diseases commonly occur in young children.

Strength of the study is that it provides health care providers key information regarding the MIH status among 7-9 years old School children in Muradnagar. There are certain limitations in this study. Since, the study variables investigated in our study depended upon mother’s interview, the possibility of recall bias cannot be denied. To minimize recall bias, an extensive questioning was performed and mothers were not informed about the possible etiological effects of the questioned disease on the MIH formation. However, the acquired data will not completely reflect the child’s medical history in his/her first three years of life. Child`s health data from medical records present in health center, will help in getting the more accurate and precise information since, this will be difficult if not impossible to achieve for all subjects in a retrospective study and illustrates the need for prospective studies. The questionnaires used in our study was close ended, thus the information obtained was limited.

## CONCLUSION AND RECOMMENDATIONS

Overall prevalence of Molar Incisor Hypomineralization in this study was 21.4%. Prevalence of Molar Incisor Hypomineralization was higher among 7-8 years age group (42.1%) as compared to 8-9 years age group (11.9%). This showed that prevalence of MIH was increasing due to unawareness of risk factors. Thus, we can conclude that MIH affects 2 out of every 10 children examined which is higher than observed in other study on Indian children. Hence, it appears that this condition is more prevalent than was recognized until recently.

Clinically MIH is a serious problem for both, affected children and dentists. These teeth are hypersensitive to cold and hot temperatures and cause mild to severe pain. Therefore, the behavioral guidance should be provided. A follow-up and recall programme for children who are affected with MIH for developing preventive and therapeutic measures and formulating public awareness and prevention programs for further control of MIH cases is more essential.

Risk Factors causing the MIH defect should be disclosed to the public. Proper treatment options should be developed for children affected with MIH, who undergo treatment more frequently than those unaffected. The general practitioner should be trained in such a way that they should have the ability to recognize MIH defects in order to perform an adequate treatment or to refer complicated cases for specialized treatment. Since children with health problems firstly go for medical treatments rather than for their dental needs, pediatricians have major role in informing parents about possible dental defects and referring them to a dentist.

## Figures and Tables

**Graph (1):**
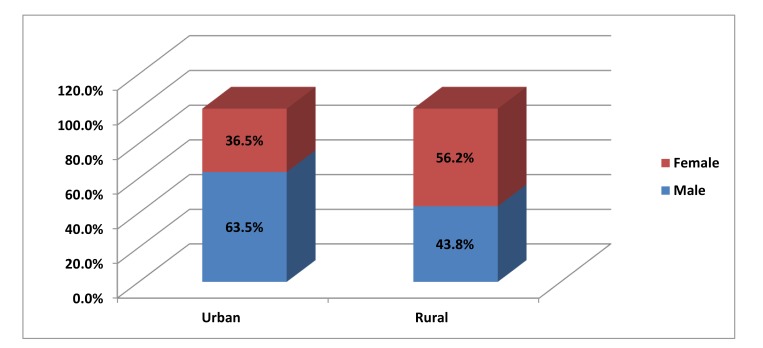
Distribution of study population according to location of school and gender.

**Graph (2):**
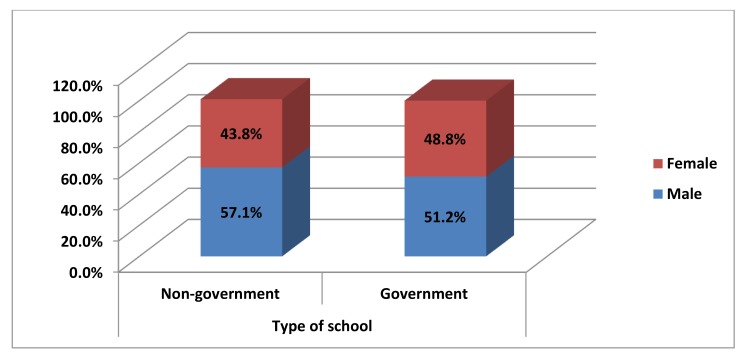
Distribution of study population according to type of school and gender.

**Graph (3):**
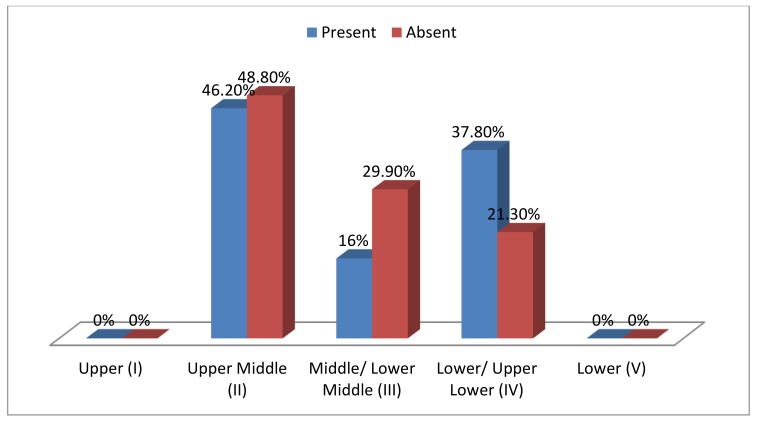
Distribution of molar incisor hypomineralization among study population according to socio-economic status.

**Graph (4):**
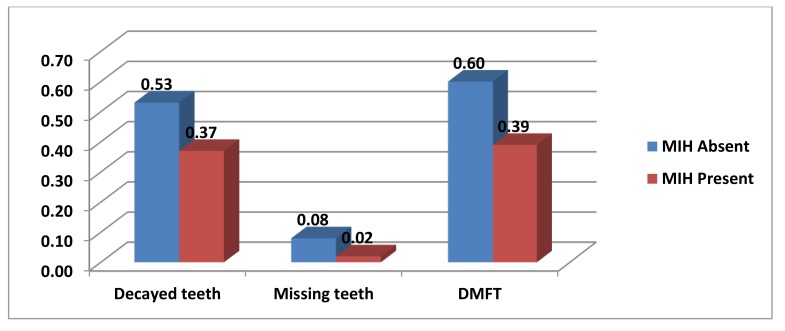
Comparison of mean of decayed teeth, missing teeth and DMFT among children with and without molar incisor hypomineralization.

**Table 1:** Distribution of study population according to age and gender.

Age (years)	Sex				Total	
	Male		Female		–	–
	Number of Subjects	Percentage Distribution (%)	Number of Subjects	Percentage Distribution (%)	Number of Subjects	Percentage Distribution (%)
7-8	150	48.2	161	51.8	311	100
8-9	382	56.1	299	43.9	681	100
Total	532	53.6	460	46.4	992	100

**Table 2:** Logistic regression analysis for association of molar incisor hypomineralization with perinatal variables.

Study Variable		MIH Prevalence	Odd’s Ratio	*p*- value	Confidence Limit
Birth complications	Yes	0%	1.304	0.0*	1.258-1.351
	No	23.3%	–	–	–
Birth prematurity	Yes	0%	1.290	0.0*	1.246-1.335
	No	22.5%	–	–	–
Excessively prolonged labour	Yes	0%	1.305	0.0*	1.259-1.352
	No	23.3%	–	–	–
Birth delivery	Normal	28.1%	2.864	0.0*	2.025-4.052
	cesarean	12.0%	–	–	–
Place of delivery	Home	22.7%	1.153	0.4	0.849-1.564
	hospital	20.4%	–	–	–

Binary logistic regression analysis * Significant difference

## References

[1] Alaluusua S. (2010). Aetiology of molar-incisor hypomineralisation: A systematic review.. Eur. Arch. Paediatr. Dent..

[2] Clarkson J. (1989). Review of terminology, classifications, and indices of developmental defects of enamel.. Adv. Dent. Res..

[3] Jälevik B., Norén J.G. (2000). Enamel hypomineralization of permanent first molars: A morphological study and survey of possible aetiological factors.. Int. J. Paediatr. Dent..

[4] Smith B.H., Kelley M.A., Larsen C.S. (1991). Advances in dental anthropology..

[5] Beentjes V.E.V.M., Weerheijm K.L., Groen H.J. (2000). A match-control study into the aetiology of hypomineralised first permanent molars.. Eur. J. Paediatr. Dent..

[6] Jalevik B., Klingberg G., Noren J.G., Barregard L. (2000). Epidemiological study of idiopathic enamel hypomineralisation in permanent first molars.. Eur. J. Paediatr. Dent..

[7] Leppaniemi A., Lukinmaa L., Alaluusua S. (2000). Nonfluoride hy-pomineralisation in permanent first molars.. Eur. J. Paediatr. Dent..

[8] Weerheijm K.L., Groen H.J., Beentjes V.E.V.M. (2000). Prevalence in 11- year-old Dutch children of cheese molars.. Eur. J. Paediatr. Dent..

[9] Weerheijm K.L. (2003). Molar incisor hypomineralisation (MIH).. Eur. J. Paediatr. Dent..

[10] Fagrell T.G., Lingström P., Olsson S., Steiniger F., Norén J.G. (2008). Bacterial invasion of dentinal tubules beneath apparently intact but hypomineralized enamel in molar teeth with molar incisor hypomineralization.. Int. J. Paediatr. Dent..

[11] Chawla N., Messer L.B., Silva M. (2008). Clinical studies on molar-incisor-hypomineralisation part 2: Development of a severity index.. Eur. Arch. Paediatr. Dent..

[12] Jälevik B., Klingberg G., Barregård L., Norén J.G. (2001). The prevalence of demarcated opacities in permanent first molars in a group of Swedish children.. Acta Odontol. Scand..

[13] Soviero V., Haubek D., Trindade C., Da Matta T., Poulsen S. (2009). Prevalence and distribution of demarcated opacities and their sequelae in permanent 1^st^ molars and incisors in 7 to 13-year-old Brazilian children.. Acta Odontol. Scand..

[14] Clarkson J., O’Mullane D., Modified D.D.E. (1989). A modified DDE Index for use in epidemiological studies of enamel defects.. J. Dent. Res..

[15] World Health Organization (1997). Oral health surveys: Basic methods..

[16] World Health Organization (1987). Oral Health Surveys: Basic methods..

[17] Ghanim A., Morgan M., Mariño R., Bailey D., Manton D. (2011). Molar-incisor hypomineralisation: Prevalence and defect characteristics in Iraqi children.. Int. J. Paediatr. Dent..

[18] Arrow P. (2008). Prevalence of developmental enamel defects of the first permanent molars among school children in Western Australia.. Aust. Dent. J..

[19] Anjum R., Sudhan Z.A. (2015). Prevalence of Molar incisor Hypomineralisation (MIH) in a group of children coming to Indira Gandhi Govt. Dental College, Jammu.. Int J clin cases Investigat.

[20] Ahmadi R., Ramazani N., Nourinasab R. (2012). Molar incisor hypomineralization: A study of prevalence and etiology in a group of Iranian children.. Iran. J. Pediatr..

[21] Zawaideh F.I., Al-Jundi S.H., Al-Jaljoli M.H. (2011). Molar incisor hypomineralisation: Prevalence in Jordanian children and clinical characteristics.. Eur. Arch. Paediatr. Dent..

[22] Shrestha R., Upadhaya S., Bajracharya M. (2014). Prevalence of molar incisor hypomineralisation among school children in Kavre.. Kathmandu Univ Med J (KUMJ).

[23] Robles M.J., Ruiz M., Bravo-Perez M., González E., Peñalver M.A. (2013). Prevalence of enamel defects in primary and permanent teeth in a group of School children from Granada (Spain).. Med. Oral Patol. Oral Cir. Bucal.

